# Strain‐Multiplex Metalens Array for Tunable Focusing and Imaging

**DOI:** 10.1002/advs.202003394

**Published:** 2021-01-04

**Authors:** Rajib Ahmed, Haider Butt

**Affiliations:** ^1^ School of Engineering University of Birmingham Birmingham B15 2TT UK; ^2^ Stanford School of Medicine Palo Alto CA 94304 United States; ^3^ Department of Mechanical Engineering Khalifa University Abu Dhabi P.O. 127788 UAE

**Keywords:** adaptive focusing, lenses, light diffraction, metasurfaces, numerical aperture

## Abstract

Metalenses on a flexible template are engineered metal‐dielectric interfaces that improve conventional imaging system and offer dynamic focusing and zooming capabilities by controlling the focal length and bandwidth through a mechanical or external stretch. However, realizing large‐scale and cost‐effective flexible metalenses with high yields in a strain‐multiplex fashion remains as a great challenge. Here, single‐pulsed, maskless light interference and imprinting technique is utilized to fabricate reconfigurable, flexible metalenses on a large‐scale and demonstrate its strain‐multiplex tunable focusing. Experiments, in accordance with the theory, show that applied stretch on the flexible‐template reconfigurable diffractive metalenses (FDMLs) accurately mapped focused wavefront, bandwidth, and focal length. The surface relief metastructures consisted of metal‐coated hemispherical cavities in a hexagonal close‐packed arrangement to enhance tunable focal length, numerical aperture, and fill factor, FF ≈ 100% through normal and angular light illumination with external stretch. The strain‐multiplex of FDMLs approach lays the foundation of a new class of large‐scale, cost‐effective metalens offering tunable light focusing and imaging.

## Graphical abstract

To tune bandwidth and focusing property, strain‐multiplexing capability of flexible‐template reconfigurable diffractive metalenses is demonstrated. A single‐pulsed, maskless light interference and imprinting technique is used to fabricate metalenses on a large‐scale. Thus, surface relief metalenses consist of meal‐coated hemispherical cavities in a hexagonal close‐packed arrangement to enhance tunable focal length, numerical aperture, and fill factor, FF ≈ 100%.

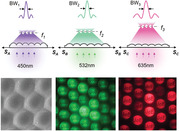

## 1 Introduction

Optical metasurfaces are engineered metal‐dielectric nanostructures on a flexible‐template with a reduced dimension that exhibit exceptional abilities to control light propagation and offer tunable opto‐mechanical capability, and larger photonic density of states from spatially arranged meta‐atoms.^[^ ^1^ ^]^ These meta‐dielectric interfaces of metasurfaces are able to manipulate light amplitude, phase, and polarization properties and show unconventional photonic behaviors^[^ ^2^ ^]^ such as such as negative reflections,^[^ ^3^ ^]^ bi‐anisotropy,^[^ ^4^ ^]^ optical clocking,^[^ ^5^ ^]^ and perfect or supperlensing.^[^ ^6^ ^]^ Therefore, optical metasurfaces are being considered as an emerging platform for multifunctional optical devices such as lenses,^[^ ^7^ ^]^ diffuser,^[^ ^8^ ^]^ beam deflectors,^[^ ^9^ ^]^ and holograms.^[^ ^10^ ^]^ Plasmonic holographic metasurfaces allow topological free‐electrons and controllable point‐excitation to generate light with prescribed wavelength, divergence and deflection or direction.^[^ ^11^ ^]^ Reconfigurable or intelligent metasurfaces allow dynamic and real‐time control of electromagnetic (EM) waves and are capable of dynamic‐functionality under the external stimuli.^[^ ^7^ ^]^ Flexible‐templated reconfigurable metasurfaces have shown tunable optical properties through applied nanomechanical strain/stretch.^[^ ^12^ ^]^ Further, strain engineering of optical and nanomechanical properties of reconfigurable flexible metasurfaces have attracted considerable attention due to extreme confinement of electrons with unique dielectric properties, that can be controlled by external fields.^[^ ^13^ ^]^ Most of these metasurface properties are structure dependent^[^ ^14^, ^15^ ^]^ and fabrication techniques are based on electron‐beam (e‐beam) lithography,^[^ ^16^ ^]^ nanoimprinting,^[^ ^17^ ^]^ template stripping,^[^ ^18^ ^]^ self‐assembly,^[^ ^19^ ^]^ colloidal synthesis,^[^ ^20^ ^]^ and nanosphere/ion‐beam milling approaches.^[^ ^21^ ^]^ However, the ability to fabricate cost‐effective, flexible‐templated reconfigurable metasurfaces, on a large‐scale for efficient opto‐mechanical tuning, at sub‐wavelength resolution, remains a great challenge.

Small miniature lenses in the dimension of micrometer scale was first introduced by Dennis Gabor in 1940, as a super lens, to focus light beyond the diffraction limit.^[^ ^22^ ^]^ Dennis Gabor's super lens is found to have a wide variety of applications in optical interconnections, beam shaping, photolithography, sensing, switching, and imaging.^[^ ^23^ ^]^ For emerging applications such as self‐driving vehicles, artificially intelligent (AI) robotic eyes, light detection and ranging (LIDAR), and head‐mounted augmented reality (AR) devices, it is highly desirable to achieve dynamic imaging and sensing by adaptive optical system.^[^ ^24^ ^]^ Adaptive light focusing can be achieved with liquid crystal‐based spatial light modulators,^[^ ^25^ ^]^ fluid‐based tunable lenses,^[^ ^26^ ^]^ electro‐optical lens,^[^ ^16^ ^]^ and tunable acoustic gradient index lenses.^[^ ^27^ ^]^ Flat metasurface lenses with adaptable local light scattering, amplitude and phase enhance numerical aperture (NA),^[^ ^28^ ^]^ aberrations,^[^ ^29^ ^]^ nonlinearity,^[^ ^30^ ^]^ and control of light field.^[^ ^31^ ^]^ Mechanically tunable metalenses are embedded in stretchable substrates to achieve tunable light focusing.^[^ ^32^, ^33^ ^]^ However, these metalenses are limited in size (less than mm), and add complexity to the nanofabrication of master‐holograms. Moreover, no theoretical and experimental demonstration of tunable bidirectional light focusing and bandwidth‐multiplexing, with external stretch/strain, exist till date.

Herein, we report strain‐multiplexed, close‐packed hexagonal arrangement metal‐coated microstructures concavity, on a flexible‐template, as diffractive reconfigurable metalenses (FDMLs), for adaptable light focusing. We utilized single‐pulse multi‐beam laser interface lithography, and achieved large‐scale (several centimeter in dimension) replication of the patterned surface structure through stamping or embossing process. Taking advantage of the smooth, uniform, periodic, and 2D *x*–*y* symmetry, fabricated metalenses show far‐field diffraction, and near‐field light focusing and act as diffractive elements. Strain‐multiplexed tunable light focusing was characterized through optical experiments, followed by numerical modeling. The fabricated metalenses show bidirectional tunable light focusing with adjustable numerical aperture and maximum fill factor (FF) of ≈100%. Further, angle‐dependent light diffraction and focusing were demonstrated through 0–360° rotation angles. Broadband and monochromatic light illumination on single text “UoB”, and holograms such as, “flower”, “sad‐face”, and “smiley‐face”, shows bright and uniform strain‐multiplexed imaging capability, with potential use in integrated optical systems, photoelectric devices, portable smart‐sensors and displays.

## 2 Results and Discussion

**Figure 1:**
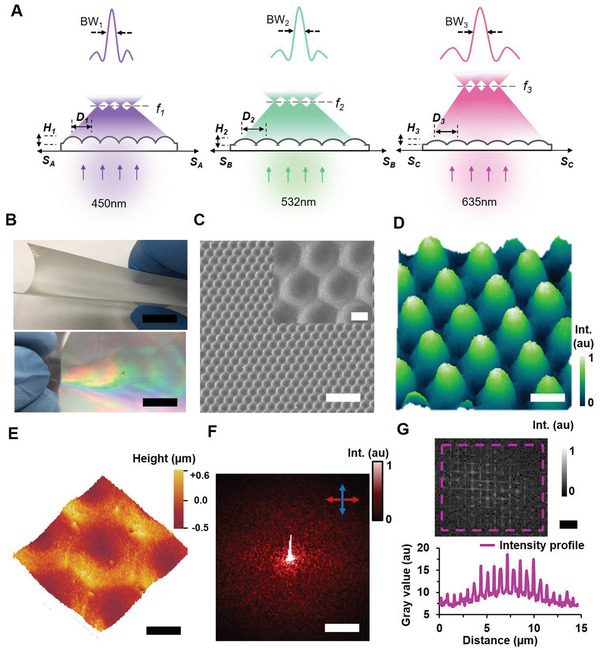
Strain‐multiplex flexible diffractive metalenses (FDMLs) and light focusing property. A) Working‐principle diagram of strain‐multiplexing and bandwidth‐tunable capability of FDMLs. B) Photograph of flexible‐template metalenses shows broadband rainbow diffraction. Scale bars = 10 cm. C) SEM and magnified image of periodic metalenses consisting of hemispherical concavity on flexible‐template. Scale bars = 10 and 2.5 µm. D) Computational modeling of three azimuth beams (exposer angle 120°) laser interference lithography. Scale bar = 3.0 µm. E) AFM surface morphology image of single metalens on flexible‐template. Scale bar = 1.5 µm. F) FFT of metalens unit cell shows light focusing. Scale bar = 50 µm^−1^. G) Upper‐part, FFT of the fabricated large‐scale metalenses show array of light focusing. Scale bar = 2 µm^−1^. Bottom‐part, Focused light intensity profile of the fabricated flexible‐templated metalenses.

FDMLs can be designed on a transparent, stretchable or flexible metasurface and optical light focusing, wavefront, and bandwidth can be tuned by external mechanical stretch/strain force (**Figure** **1**A).^[^ ^13^ ^]^ The stretching of the flexible‐template metasurface changes the light‐scattering diffractive element's relative positions and enable phase discontinuity of the illuminated light.^[^ ^33^ ^]^ We fabricated hemispherical micro‐concavities through laser interference on a flexible‐template (Figure S1A, Supporting Information). These plasmonic metal‐coated metasurface act as an optical scattering and focusing elements to the incident light.^[^ ^14^ ^]^ These scattering elements can be varied on a wavelength‐scale through external stretch which enables wavefront shaping and provide different optical functionalities such as, adaptive light focusing or tuning. Optical phase discontinuity can be further tuned by varying the size, shape, and scattering orientation on flexible‐template metasurfaces. To understand the changes of the illuminated electric field profile, *E*_o_(*x*,*y*) = *A*e^*ik* *ϕ*(*x*,*y*)^ on a stretchable metasurface, we can consider applied isothermal mechanical strain, *S* to modify *E*‐field profile approximately as*E*′_0_(*x*′,*y*′) = *E*_0_(*Sx*,*Sy*)*A*e^*ik* *ϕ*(*Sx*,*Sy*)^. Similarly, modified electric wavefront shaped by a stretchable metasurface at arbitrary distance, z′can be approximated by using well‐known Fresnel transformation,^[^ ^33^ ^]^

(1) $$E^{\prime }\left(x^{\prime },y^{\prime },z^{\prime }\right)=E\left(Sx,Sy,S^{2}z\right)=E\left(x,y,z\right)e^{ik\left(s^{2}-1\right)z}$$

here E^′^ is the modified electric field and *S* is external stretch factor. As we modify the metasurfaces with an isotopic stretching factor of *S*, the modified electric field distribution will appear at distance *S* ^2^. Similarly, as we stretch metalens array, the focal distances will vary according to the stretching factor of *S^2^*, when compared with normal/un‐stretched part of FDMLs. Further, as we increase the stretching factors of metalens array (*S*_A_ < S_C_), light scattering elements further gets displaced and contribute to increase the full width‐half‐maximum (FWHM) of the focused light. Therefore, flexible‐template metasurface lens array is able to tune light focusing property and bandwidth‐multiplexing capability. Further, these photonic surface relief nanostructures are able to produce constructive interference of selective visible wavelength and can act as color‐selective diffractive narrowband tunable filter.^[^ ^34^ ^]^

Large‐scale fabrication of FDMLs is involved in multi‐beams laser interference based micro‐patterns and multiple replications using stamping/embossing steps (Figure S1 A,B, Supporting Information). During master hologram fabrication, a positive photoresist (PR) (diazonaphthoquinone) layer on a glass plate were spin coated (thickness: 1 mm) for 2 min at 100 rpm and heated for 1 h at temperature 80 °C. Three azimuthal beams (458 nm wavelength, 120° exposer angle, and 100 mW optical power) interference on the PR layer for 1 min. Azimuthal beams interface results in hemispherical surface structure on PR layer in hexagonal close‐packed arrangement.^[^ ^34^ ^]^ The interference process was repeated until the entire PR layer was laser exposed. PR developer was used to remove exposed area and to create FDMLs. Finally, a master hologram was created by evaporating nickel layer (300 nm) on patterns PR surface. Multiple copies were replicated from the master hologram using ultraviolet (UV) nanolithography onto acrylate polymer (AP) (2 µm) over a poly(ethylene terephthalate) (PET) (100 µm) substrate. Further, silver metal‐layer (3nm) is coated over AP (details available in the Experimental Section). Photograph images of FDMLs show flexibility, semi‐transparency, and broadband rainbow diffraction from the fabricated surface patterns (Figure 1B). The scanning electron microscope (SEM) image of samples shows hemispherical structures in hexagonal arrangement and periodic surface profile (Figure 1C; Figure S2A,B, Supporting Information). Surface profile of FDMLs structure ensures ≈3.4 µm lateral spacing (diameter, *D*). Computational 3D modeling of three azimuth beams (120° illumination angles) interference also shows hexagonal intensity patterns (Figure 1D). Atomic force microscopic (AFM) image of the single FDMLs shows morphological feature (Figure 1E). AFM feature of the FDMLs structure having ≈1.1 µm height (*H*) variation. Light focusing property of the fabricated FDMLs was ensured with numerical computation before performing optical characterization. First Fourier Transfer (FFT) of the fabricated sample shows single and array light focusing property (Figure 1F,G; Figure S2C, Supporting Information). FFT of single FDMLs structure shows light focusing at the center and symmetrical intensity distribution along *x*‐ and *y‐*directions (Figure S2D, Supporting Information). FFT of large scale FDMLs sample ensure multiple peaks in the intensity profile which indicates that fabricated FDMLs have efficient light focusing property (Figure 1G). Further, multiple focused light intensity of FDMLs sample was also observed through an optical microscope (Figure S2E, Supporting Information).

**Figure 2:**
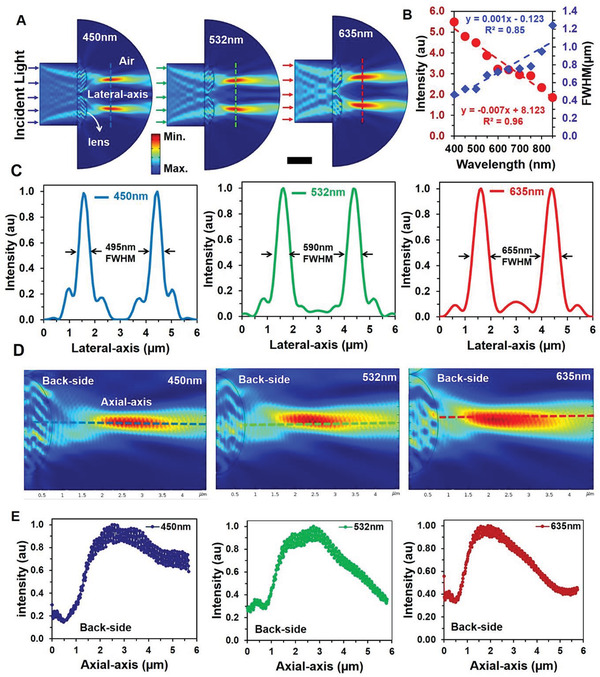
Numerical modeling of FDMLs light focusing along lateral and axial‐axis. A,B) Monochromatic field distribution of back‐side light focusing of FDMLs. Scale bar = 3.3 µm. C) Focused light intensity profile and FWHM as a function of wavelength (635, 532, 450 nm). D) E‐field intensity distribution of unite FDMLs along lateral‐axis with monochromatic light illumination. Scale bar = 0.75 µm E) Normalized focused intensity profile of unite FDMLs along axial‐axis with monochromatic light illumination.

To understand optical light focusing capability of FDMLs, numerical modeling was computed using COMSOL Multiphysics tool (**Figure** **2**). The computed FDMLs model consisted of hemispherical structure with periodicity 3.4 and height 1.1 µm. The hemispheres were made with AP on PET film. The bulk refractive index of AP and PET substrate were defined as 1.49 and 1.57, respectively.^[^ ^34^ ^]^ A monochromatic light was illuminated from the back‐side and light focusing capability of FDMLs structure was observed at the other side of hemispherical periodic structures (Figure 2A). Focused field intensity distribution was observed with monochromatic light at normal illumination. As wavelength increased, FWHM or 3dB bandwidth (BW) increased at focal point (Figure 2B). However, focused light intensity values decreased as monochromatic wavelength increased. A linear shift of FWHM and focused light intensity values were observed as incident wavelength increased. Well‐order symmetric light focusing and reduced FWHM at focal point were observed with monochromatic red, green and violet (632, 532, and 450nm) light at normal illumination (Figure 2C). Lateral and axial‐axis observation planes were placed perpendicular to the focused light to observe electric field (*E*‐field) intensity distribution (Figure 2D). Light focusing along axial‐axis showed wider response compared with vertical axis (Figure 2E). Similar to back‐side, front‐side incident light illumination and optical focusing property of FDMLs was also observed. Therefore, FDMLs show bidirectional light focusing due to front‐side (hemispherical surface is opposite to illumination) and back‐side (hemispherical surface is infornt of light illumination) light illumination (Figure S3, Supporting Information). Back‐side focusing property of FDMLs also shows efficient light focusing along lateral and axial‐axis (Figures S4–S6, Supporting Information). Moreover, back‐side focusing shows narrower 3 dB bandwidth when compared with front‐side focusing and is more suitable for bright focusing with high precision and details image analysis.

**Figure 3:**
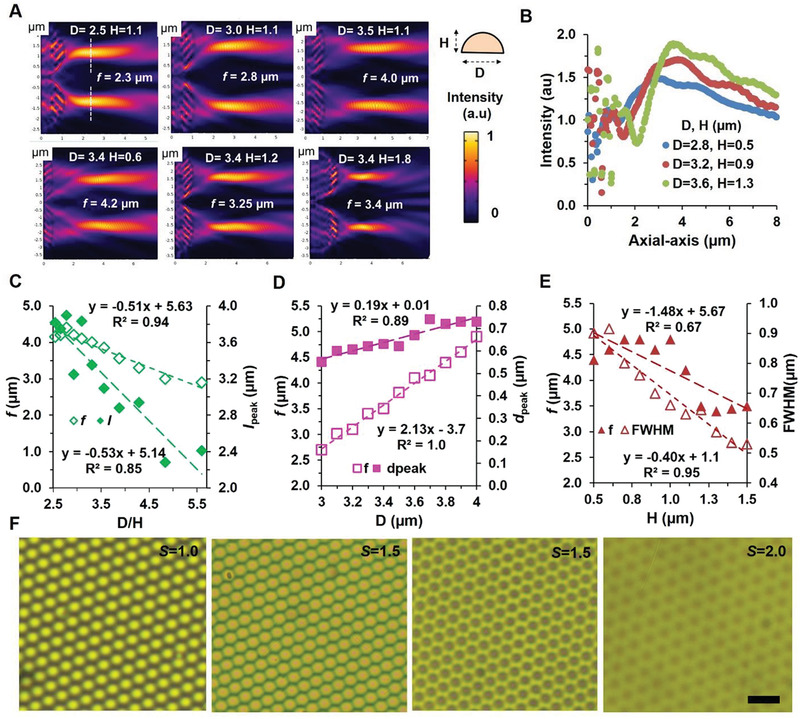
Strain‐multiplex tunable light focusing property of flexible‐templated FDMLs. A) Focused E‐field intensity distribution as a function of diameter (*D*) and height, (*H*), variation. Scale bar = 1.0 µm. B) Intensity distribution (along axial‐axis) of the focused light with *D* and *H* variation. C–E) The bandwidth (FWHM) and peak‐to‐peak distance (*d*_peak_) of focused light as a function of width/height variation. F) Optical microscopic images of strain‐multiplex light focusing property with applied stretch factor of *S* = 1, 1.5, 2.0 and 2.5. Scale bars = 10 µm.

Tunable light focusing property of FDMLs is related to the applied external strain force or stretch. If FDMLs stretched with a factor of *S*, the focused electromagnetic field will be appear at distance *f′ = S* ^2^ *f*, where *f* is the original focal length of unstretched flexible metasurface.^[^ ^32^ ^]^ New focused electric field distribution also expand with a factor of *S* compared with the unstretched one. Therefore, structural parameters of FDMLs (diameter, height or their accept ratio) will change due to external stretched factor of *S*, provide tunable optical performance and modified lens parameters that can be approximated by using the following relations,^[^ ^35^, ^36^, ^37^ ^]^

(2) $$\begin{array}{c} R^{\prime }=SR=S\left[\left(H^{2}+r^{2}\right)/2H\right]\\ f^{\prime }=S^{2}f=S^{2}\left[R/\left(n_{\mathrm{eff}}-1\right)\right]\\ {f^{\prime }}_{\#}=Sf_{\#}=S\left[f/2r\right]\\ and\;{\mathrm{NA}}^{\prime }=\mathrm{SNA}=S^{-1}\left[D/2f\right] \end{array}$$

where *R* is the radius of curvature, *f* is the focal length, *f*_#_ is the *f*‐number, *n* is the refractive index of the materials, and NA is the numerical aperture of the unstretched FDMLs structures (*S* = 1). Further, we have *d* = 2*r* ≈ 3.4 µm, *H* ≈ 1.1 µm, *n*_AP_ = 1.49 for AP, *n*_PET_ = 1.57 for PET, *n*_eff_ = 1.56 for AP‐PET substrate. Therefore, calculated lens parameters are *R* = 1.864, *f =* 3.3 µm*, f*_#_ = 1.0, and NA = 0.5, respectively. FWHM of diffraction limited spot size can be calculated as*λ*/(2 × NA) where *λ* is the designed or operating wavelength, NA is the numerical aperture, and FWHM is the full width‐half‐maximum of diffraction limited spot size.^[^ ^38^ ^]^ For FDMLs, we have found FWHM of diffraction limited spot size ≈1.1 times higher compared with theoretical limit (airy profiles). Further, lens parameters are tunable with external stretch factor of *S*. During fabrication, the width/height aspect ratio of the FDMLs can vary with laser flounce, exposer time or pulse duration. Improved imaging quality strongly depends on *R* value and depends on width and height aspect ratio of lens geometric structure. Further, higher NA value are highly desired for improve image quality which is also a function of *R* value. Further, light focusing capability of FDMLs is strongly dependent on structural parameters (diameter and height) variation due to applied external stretch factor of *S*. Structural parameters are able to control light propagation direction and enable constructive interface of incident light at FDML's focal point. Finally, FDMLs with desired performance parameter enable improved image quality. **Figure** **3**A shows FDMLs light focal point variation with diameter (*D*), and height (*H*) variation. Tunable focal length, *f* is observed, if we keep fixed *h* = 1.1 µm, and varied *D* = 2.5, 3 and 3.5 µm, respectively. Similarly, tunable *f* is observed, if we keep fixed *D* = 3.0 µm, and varied *H* = 1.0, 1.25, and 1.4 µm, respectively. As *D* and *H* of FDMLs increases, the focal point, *f* is moved to the higher and lower positions. Similarly, if we varied the *D* and H simultaneously, *f*‐values move to the higher values as observed in Figure 3B. The spot size or FWHM of focused light becomes wider as we increase *D* and *H* values (Figure 3C). The peak‐to‐peak distance (*d*_peak_) is also increased as we increase *D* and *H* values (Figure 3D). Moreover, if we increase ratio of *D*/*H* values, FWHM values increase linearly. However *d*_peak_ values are decreased linearly as a function of *D*/*H*. Similarly, the focal length (*f*) and peak intensity (*I_peak_*) are linearly decreased as a function of *D*/*H* (Figure 3D). Further studies were performed to observe optical property of the focused light through *D* and *H* variation. For *D* (fixed *H* = 1.1) variation, the focal length (*f*) and peak‐to‐peak distance (*d*_peak_) are increased linearly (Figure 3D). Similarly, FWHM and *d*_peak_ also increased linearly with *D* variation (Figure S6A,B, Supporting Information). For *H* (fixed *D* = 3.4) variation, *f* and FWHM values are decreased linearly (Figure 3D). Similarly, focused light intensity also increased linearly with *H* variation (Figure S7B, Supporting Information). As *H* value increased, the focal length, *f* moved to lower values as observed in Figure 3E. Additionally, as we increase the ratio of *D*/*H*, we observed that focused focal length, *f* decreases (Figure S7C, Supporting Information). The performance parameters of FDMLS show dependence on lens structural parameters (diameter, height) and their aspect ratio, which were also previously reported.^[^ ^35^, ^36^ ^]^ Further, we have also measured tunable light focusing of FDMLs with *D*/*H* variation due to no stretch (*z ^´^* = *f*) and external stretch factor of *S* = 1.5, 2.0, and 2.5 (Figure 3F). As we increase *S* value, focal point moves to higher unfocused positions (*z′* = *f* + *z*) and light intensity decreased.

**Figure 4:**
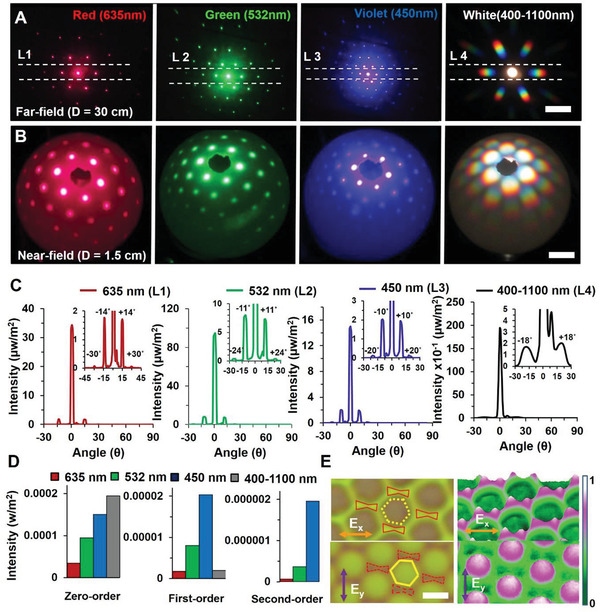
Far‐ and near‐field light diffraction, focusing and polarization properties of FDMLs. A,B) Experiment results for the far‐ and near‐field light diffraction and focusing of FDMLs through an image‐screen and transparent hemispherical surface. Hexagonal diffraction of FDMLs through broadband (400–1100 nm) and monochromatic (635, 450, and 450 nm) light illumination. Scale bars = 8 and 2 cm. C,D) Measured focused light diffraction intensity of FDMLs as a function of angular rotation and diffraction orders through a broadband and monochromatic light illumination. E) Optical microscopic images of FDMLs with polarization sensitive hot‐spots areas. Scale bars = 2.5 µm.

Far‐field and near‐field light diffraction and focusing from FDMLs were observed with an image screen and hemispherical experiment (**Figure** **4**A,B; Figure S9 A,B, Supporting Information). As monochromatic light (635, 532, and 450 nm) illuminated the FDMLs sample, higher order diffraction patterns are visualized. Higher‐order light diffraction distance from the non‐diffractive zero‐order was increased with higher wavelengths. Therefore, maximum and minimum light diffraction distance from zero‐order was observed with red and violet light illumination. Rainbow diffraction pattern was also observed with broadband white light illumination. Like monochromatic wavelength, red band of the rainbow patterns diffract more compare with violet band. Efficient bidirectional diffraction of FDMLs was observed with front and back‐sider normal illumination of monochromatic and broadband light. Each FDML is focused light at the center of the hemisphere in a hexagonal arrangement. This is due to the reason that fabricated hemispheres of FDMLs are in hexagonal arrangement and act as a diffractive FDMLs. Hexagonal 2D light diffraction was also observed with near‐field through monochromatic (red, green and violet) and broadband light illumination. As previously, higher‐order diffraction distance is observed with higher wavelength values. Light diffraction from periodic FDMLs can be explained with Bragg's law, *Λ* = *λ*/2sin*ϴ*, where *Λ* is the hemispherical spacing, *λ* is the incident wavelength, and *ϴ* is the light illumination angle. Furthermore, angle resolve diffraction of FDMLs was measured with 3D rotational measurement.^[^ ^39^ ^]^ Sample holder in the *x*–*y* stage was used to fix FDMLs. A broadband and monochromatic light were normal illuminated to the sample and diffracted light was measured through an optical spectrometer. Light source and spectrometer are in same line to the sample holder but in opposite positions. The FDMLs sample and light source rotated with a steeper motor from 0 to 180° (1° step size) to measure diffracted light intensity. Light diffraction from FDMLs was measured as a function of angular rotation (Figure 4C). Higher‐order light diffraction angle from non‐diffractive zero‐order increased as a function of wavelength. Therefore maximum and minimum angular light diffraction distances were observed with monochromatic red and violet light illumination. A wide band rainbow diffraction was measured at 18° angular diffraction distance from non‐diffracted zero order through broadband illumination. Diffraction orders increased with monochromatic wavelength. Therefore, blue light shows maximum diffraction order when compared with red light. Maximum and minimum light diffraction intensity were observed with green and violet monochromatic light illumination (Figure 4D).

Light diffraction performance through broadband and monochromatic illumination on the FDMLs are comparable with other reported structures fabricated with direct laser and embossing process.^[^ ^40^ ^]^ Diffractive focusing property of FDMLs was based on constructive interference of incident light beams and having dependence with incident wavelength and structural parameters such as *D* and *H* variation. Diffraction distance is increased for monochromatic and broadband illumination like other periodic hemispherical surfaces patterns.^[^ ^41^ ^]^ Optical diffraction property of FDMLs was also numerically modeled (Figure S8, Supporting Information). These FEM based simulation ensure well‐ordered light diffraction with red, green and violet monochromatic light illumination (Figure S8B, Supporting Information). Higher‐order diffraction distance from non‐diffracted zero‐order was increased with higher monochromatic wavelength. Therefore, maximum (6th‐order) and minimum (3rd‐order) light diffraction order were observed with red and violet light at normal illumination. Diffracted light diffraction angles were decreased with lower illumination wavelengths. Further, polarization dependent hot spots were observed at the edge and center of microstructures through broadband light illumination (Figure 4E). At *x*‐polarization, hotspots were observed at the boundary/edge sides of FDMLs. However, edge hotspots are disappears at *y*‐polarization light and center hotspots are observed at the middle of FDMLs. Polarization dependent 3D images of the hotspots ensure further details (Figure S8C, Supporting Information). Polarization‐dependent hotspots arise at the connections between neighboring structures due to plasmonic resonance excitation by polarized‐light wave.^[^ ^42^ ^]^ Therefore, the enhancement of localized electric and magnetic fields is observed based on polarizations.^[^ ^43^ ^]^ This polarization‐dependent hotspots and optical properties were previously demonstrated at sub‐wavelength scale nano/microstructures by using numerical platform and followed with experimental validation.^[^ ^42^, ^43^ ^]^ Furthermore, polarization‐dependent hotspots are of interest in different applications including bio‐sensing, precise light manipulation and a simplified route for polarization imaging.^[^ ^44^, ^45^ ^]^ The metalenses with birefringent meta‐atoms showed polarization‐controlled multifunctional properties and broadband achromatic focusing.^[^ ^46^ ^]^ This polarization‐multiplexing capability enabled broad phase dispersion coverage and will be suitable for a large‐scale and high performances metadevices.

**Figure 5:**
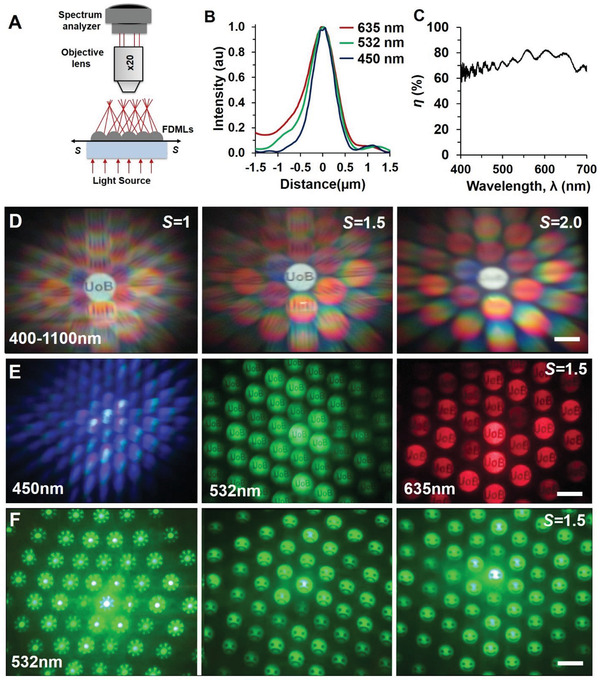
Strain‐multiplex light focusing of FDMLs. A,B) Optical setup and light focusing property through a monochromatic (635, 532, and 450 nm) light at normal illumination. C) Focused light efficiency, *η* as a function of broadband (400–1100 nm) illumination wavelength. D,E) Far‐field focusing of “UoB” text image/hologram through broadband and monochromatic light at normal illumination during focused (*Z* = *f*) and stretch factor of *S* conditions. Scale bars = 5 cm. F) Far‐field focusing of “Flower”, “Sad‐face”, “Smile‐face” image/hologram through a monochromatic (532 nm) light at normal illumination during focused (*Z* = *f*) and *S* = 1.5 conditions. Scale bars = 5 cm.

Optical light focusing properties of FDMLs were further analyzed through monochromatic, broadband light illuminations, and image projection experiments (**Figure** **5**). Monochromatic and broadband light illuminated from the back‐side and focused light intensity was measured on the other‐side through a spectrum analyzer (Figure 5A). Focused light BW increased as we increased illumination wavelengths (Figure 5B). Further, we measured focused light efficiency, *η* as the percentage ratio between broadband light transmission of FDMLs and silica glass plate. Maximum focused transmitted light efficiency, *η* ≈ 83% was measured in the wavelength range of 450–700 nm (Figure 5C). We have measured focused transmitted light as a ratio between transmitted focused light with FDMLs sample and transmitted unfocused light without FDMLs sample.^[^ ^47^ ^]^ Maximum and minimum focused light intensity was approximately observed, measured at 443 nm (violet) and 557 nm (green) respectively. As the wavelength of the incident light increased, the focused light BW (FWHM) increased linearly. Further, focused light intensity level increased with higher wavelength. Therefore, the bandwidth‐multiplex color‐light focusing property of FDMLs is observed at wavelength‐scale. Further, in the strain‐multiplex image projection experiment, the text and hologram are illuminated with a broadband and monochromatic light (Figure 5D–F). FDMLs sample are placed after the hologram and passed through an optical objective (20×). Finally text and hologram images are focused and captured by a far‐field image screen setup (Figure S9C, Supporting Information). FDMLs sample act as a miniature lens array that is able to focus illuminated text or hologram and show multiple copies at the image screen. Far‐field image project experiment of FDMLs did well with front and back‐side illumination. The bidirectional light focusing property of FDMLs was observed due to symmetrical surface structures. Moreover, efficient light focusing was observed due to Bragg's effect from the hemispherical surface‐relief microstructures. Diffracted light from the hemispherical microstructures focused at the focal point due to constructive light interference. For broadband light illumination at “UoB” mask, far‐field focused (*z*_0_ = *f*) at no stretched (*S* = 1) and unfocused (*z* = *f+ z*_0_) with applied stretched factor of *S* = 1.5 and 2.0 conditions through image projection are illustrated. Color‐full rainbow diffraction patterns of “UoB” text are observed into the image screen during no stretch (focused) and stretched (unfocused) conditions. Further, monochromatic light (red, green and violet) is illumination to the “flower”, “sad‐face”, and “smiley‐face” holograms at stretched factor of *S* = 1.5. The far‐field captured images are of similar size and shape due to the precise and uniform structure of the fabricated FDMLs that enable high‐resolution imaging.

## 3 Conclusion

We have reported a plastic‐template flexible hemispherical surface‐relief microstructure as FDMLs and shown efficient stain‐multiplex bidirectional focusing. The FDMLs structures are fabricated with multi‐beams laser interference lithography and imprinted into multiple copies through stamping/embossing process. The strain‐multiplex optical focusing and light diffraction properties of fabricated FDMLs are experimentally demonstrated followed with computational modeling. Light diffraction performance through broadband and monochromatic illumination on the FDMLs are comparable with other reported structures fabricated with direct laser and embossing process.^[^ ^40^ ^]^ Light focusing property of FDMLs was based on constructive interference of incident light beams and having dependence with incident wavelength and structural parameters such as width and height variation. As the wavelength of the incident light increased, the focused beam width (FWHM) increased linearly. However, focused light intensity level decreased linearly with higher wavelength. The performance parameters of FDMLs show dependence on applied strain factor, *S* due to lens structural parameters (diameter, height) and their aspect ratio, which were also previously reported.^[^ ^36^, ^44^, ^48^ ^]^

Optical microscopic arrangement ensure broadband and monochromatic light focusing at focal distance *f* = 3.4 µm, which was similar to computational model. Tunable *f*‐number, *f*, and NA can be achieved with the variation of stretched factor of *S*, due to change of radius of curvature, *R* which is also a function of *D* and *H*. Further, structural parameters (*D*, *H*) can be controlled through laser parameters such as peak energy/power, exposer duration and angle. Previously reported lenses are limited with broadband and unidirectional focusing at normal illumination.^[^ ^35^, ^36^ ^]^ Herein, we report diffractive FDMLs that enable bidirectional strain‐multiplex focusing though broadband and monochromatic light at normal and angular illumination. The *f*‐number and NA are important parameters used to define light gathering capability of FDMLs, where lower *f*‐number and higher NA are desired to achieve maximum light gathering and resolving specimen details for bright and dark imaging.^[^ ^49^ ^]^ Another important performance parameter of FF can be define as a ratio between lens areas to total areas. Moreover, FF can also be defined as the number of blacked pixel and total pixel for a binary image. Circular lenses in a square and hexagonal arrangement were shown FF about 78% and 90.7%, respectively.^[^ ^48^ ^]^ However, maximum 100% FF (full) is achieved with hexagonal FDMLs in hexagonal arrangements due to full coverage area of the single cells. 100% FF of FDMLs may enable maximum light coupling efficiency of optical devices such as LEDs or organic‐LEDs.^[^ ^50^ ^]^ Finally, we anticipate that our FDMLs with strain‐multiplexing capability will enhance light‐mater interactions, and color‐selective focusing application in adaptive nanophotonic and plasmonic.

## 4 Experimental Section

##### Flexible‐Template Metalenses Fabrication and Replication

The fabrication of plastic‐template flexible FDMLs involves multi‐beams laser interference based micro‐patterns and a large‐scale replication using stamping/embossing steps (Figure S1A,B, Supporting Information). A ≈2.0 µm thickness positive PR (diazonaphthoquinone) layer was spin coated (100 rpm, 2 min) over a 1 mm glass substrate and backed for 1 h at 80 °C (Figure S1B(i), Supporting Information). Hemispherical surface patterns on the PR layer (*n* = 1.65) were performed through laser exposer steps (Figure S1a(ii)). Hexagonal arrangement of FDMLs structures were created through multi‐beams interference lithography. During laser exposer step, three azimuthal beams (exposer angle, *ϴ* = 120°, duration = 60 sec.) were illuminated in PR layer. Conceptual block diagram regarding optical steps of three azimuthal beams interference is illustrated in Figure S1A, Supporting Information. A laser beam (*λ* = 458 nm, 100 mW, *Ø* = 14 cm) was illuminated to a beam splitter (BS1, 2/3:1/3) that produce two output beams. 2/3 of input power pass that through optical path 1 (P1) and 1/3 of input power pass that through optical path 2 (P2). BS2 divide OP2 into 2 beams: each beam carry 1/3 of input power. Finally, P3, P4, P5 beams carry equal amount of input power (1/3:1/3:1/3) and pass through a series of pinholes, partial filters, and lenses. The beams P4, P5 pass through optical phase‐shifters (FS) to produce 120° phase‐difference with P3. Finally, three azimuthal beams (exposer angle: 120°) produce interference onto the target materials (PR samples). Surface patterns produces in a hexagonal arrangement due to lateral and horizontal interference patterns of azimuthal beams (Figure S1B(iii), Supporting Information). A photographic developer was used to etch PR patterns to produce FDMLs (Figure S1B(iv), Supporting Information). Silver and nickel maser hologram of PR nanostructures was created using electroplating process (Figure S1B(v–vi), Supporting Information). Finally, a master hologram was used to create replication of FDMLs nanostructures on AP‐PET substrate using UV stamping/embossing process (Figure S1B(vi), Supporting Information). Replicated holograms shows hemispherical structures in a hexagonal arrangement (Figure S1B(vii), Supporting Information). Finally, replicated AP‐PET surface was coated with ≈3nm silver layer through an evaporation process to increase light diffraction and act as a scattering element for metalenses (Figure S1B(viii), Supporting Information). Further details regarding fabrication process are available at Figure S1, Supporting Information, and literatures.^[^ ^34^, ^37^ ^]^

##### Optical Modeling

Computational modeling of light diffraction and bidirectional focusing of FDMLs was modeled through FEM based commercial simulation tool (COMSOL Multiphysics v5.3, California, USA). The unit cell of model geometry consisted of two hemispherical structures with periodic, scattering, and continuity boundary conditions were placed across the periodic, outer sides, and internal structures. Further, computational environment was considered with triangular meshing elements (maximum mesh size was one‐tenth of input wave length). Computational geometry was consisted with 35800 domain elements, 1722 boundary elements, and 25162 degrees of freedom. Incident light was illuminated from the left side and focused light intensity were computed from the right side of FDMLs. Taking advantage of planar symmetric, 2D FDMLs model geometry was considered with hemisphere structure of AP (*n* = 1.49) having spacing of 3.4 µm on a PET (*n* = 1.57) substrate. A hemispherical surface filled with air (*n* = 1) medium are considered surrounding the periodic FDMLs structures. Height and width of FDMLs were varied to compute optimized focusing capability and calculated lens performance parameters such as *f*, FWHM, *I*_peak_, and *d*_peak_. Continuity and scattering boundary conditions were considered for FDMLs structure and surrounding medium. During modeling, materials property of the FDMLs structure and surrounding medium were defined from the source program.

##### Optical Characterization

Far‐field diffraction and near‐field focusing property of FDMLs were measured through light illumination at normal and angular setups. Diffracted and focused light intensity spectra were measured through an Ocean Optics spectrometer (spectrum resolution ≈ 0.1, FWHM ≈ 0.2 nm), broadband (400–1100 nm, OSL2 fiber illuminator) and monochromatic red (635 nm, 4.5 mW, Ø11 mm), green (532 nm, 4.5 mW, Ø11 mm), and blue (492 nm, 2.6 mW, Ø11 mm) illumination which are purchased from Thorlabs. Angular measurement was performed through a customized 2D –y rotational stages operated with stepper motor (minimum step size 1 degree). Far‐field image projection and diffraction experiment was performed on image screen (white A4 page) and transparent hemispherical (diameter Ø30 cm) setups.

## Conflict of Interest

The authors declare no conflict of interest.

## Supporting information

Supporting Information
