# Supplementary material for: Strain‐Multiplex Metalens Array for Tunable Focusing and Imaging
Source: Adv Sci (Weinh). 2021 Jan 4;8(4):2003394. doi: 10.1002/advs.202003394 (PMC7887606; doi:10.1002/advs.202003394)
Supplement: Supplementary file 1 — Supporting Information [file ADVS-8-2003394-s001.pdf]

WILEY-VCH

© 2020 Wiley-VCH GmbH

ADVANCED SCIENCE
Open Access

Supporting Information

for *Adv. Sci.*, DOI: 10.1002/advs.202003394

# Strain-Multiplex Metalens Array for Tunable Focusing and Imaging

*Rajib Ahmed* and Haider Butt**

# Supporting Information

# Strain-Multiplex Metalens Array for Tunable Focusing and Imaging

Rajib Ahmed and Haider Butt

Rajib Ahmed

School of Engineering, University of Birmingham, Birmingham, CA 94305, UK

Stanford School of Medicine, Palo Alto, CA 94304, United States

Haider Butt

Department of Mechanical Engineering, Khalifa University, Abu Dhabi, P.O. 127788, UAE

**Email:** rajibah@stanford.edu (R.A.), haider.butt@ku.ac.ae (H.B.)

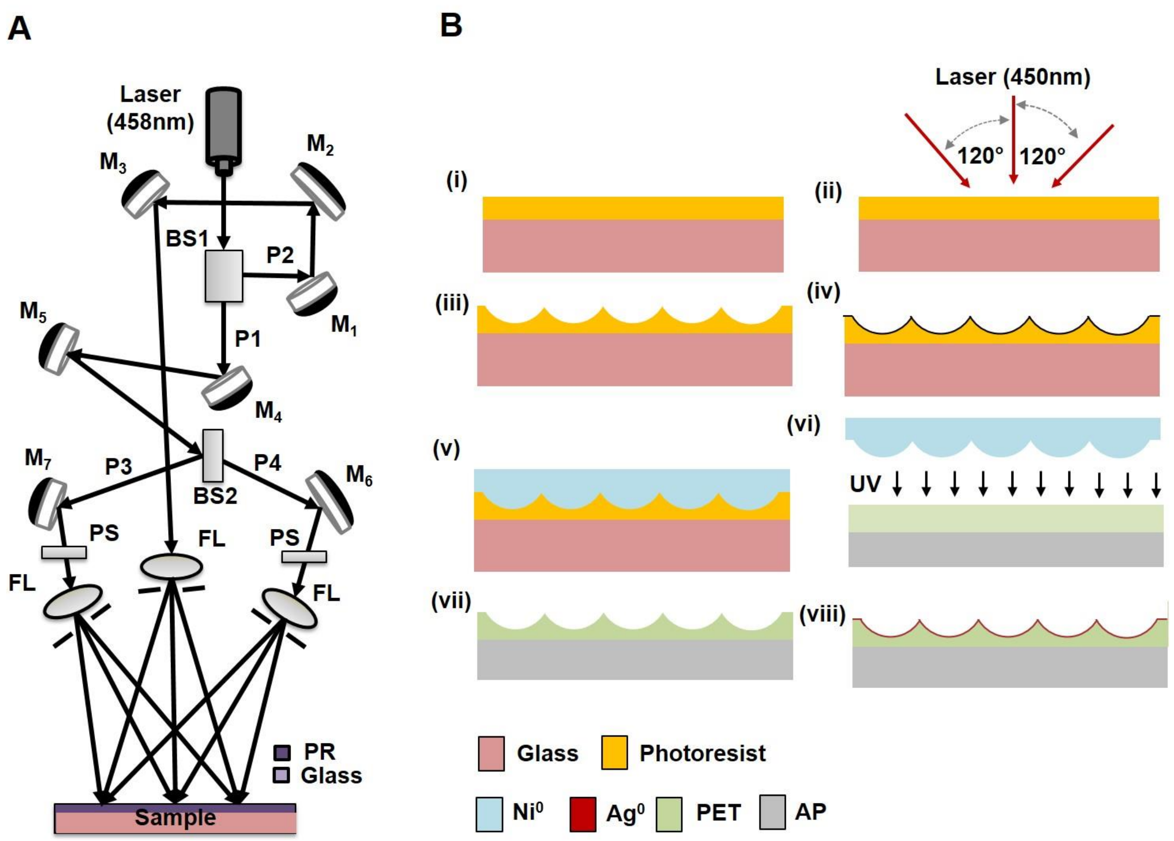


**Figure S1.** Multi-beams laser interference optical setup and replication process. (**A**) Experiment setup for the multi beams laser interference lithography for Flexible-templated diffractive metalenses (FDMLs). (**B**) Laser interface based 3D nanofabrication of FDMLs and replication steps through stamping/embossing process.

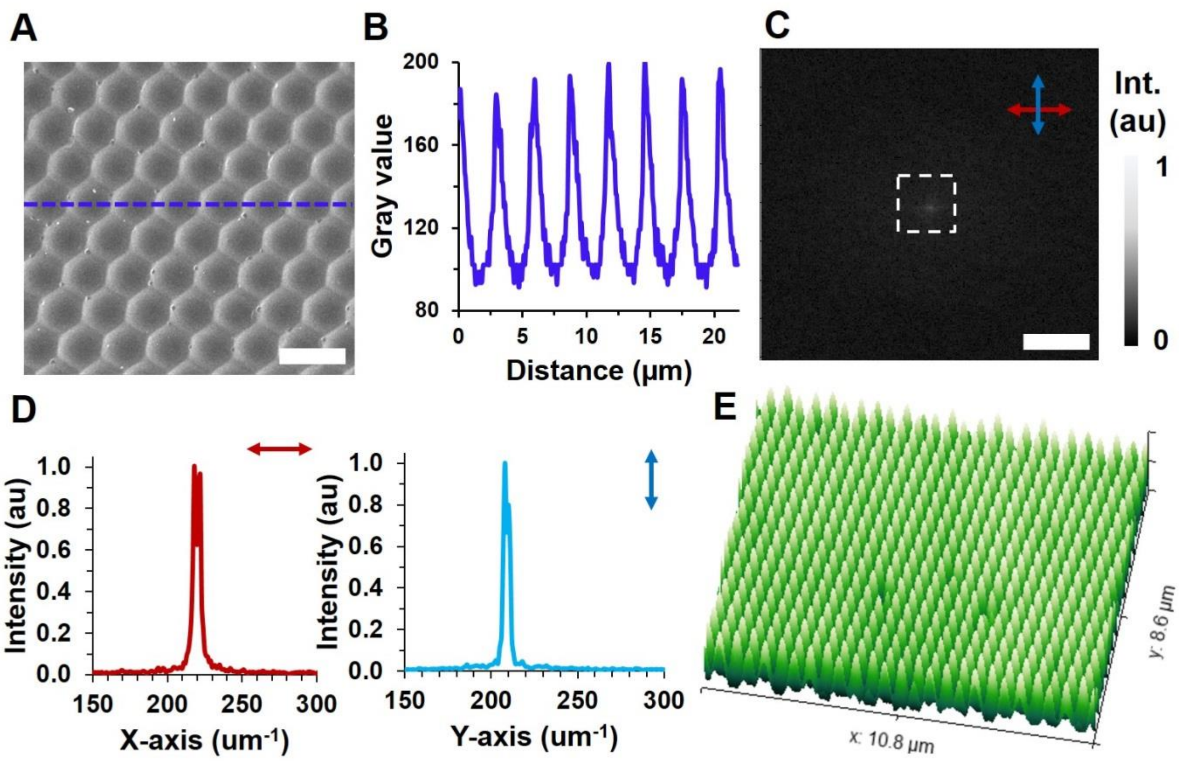


**Figure S2.** Fabricated SEM of FDMLs, surface profile, and light-focusing. **(A, B)** Fabricated sample and surface profile of FDMLs. Scale bar = 5 µm. **(C-D)** FFT of single cell FDMLs shows light focusing at the centre and symmetrical intensity distribution along x and y-directions. Scale bar = 25 µm. (**E)** Focused light property of FDMLs by using optical microscopic.

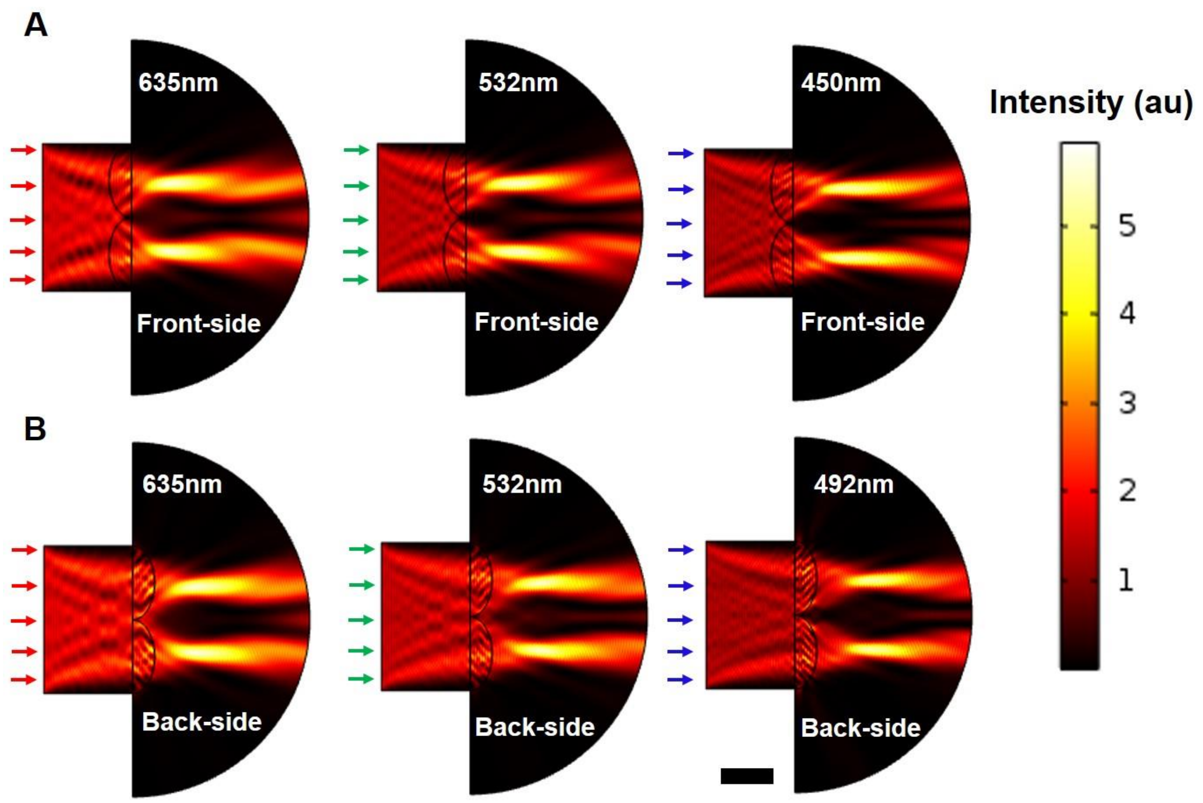


**Figure S3.** Bidirectional light focusing of FDMLs. (**A, B**) Bidirectional (front and back-side) light focusing property of FDMLs through monochromatic (635, 532, and 450 nm) light at normal illumination. Scale bar = 2.5 µm.

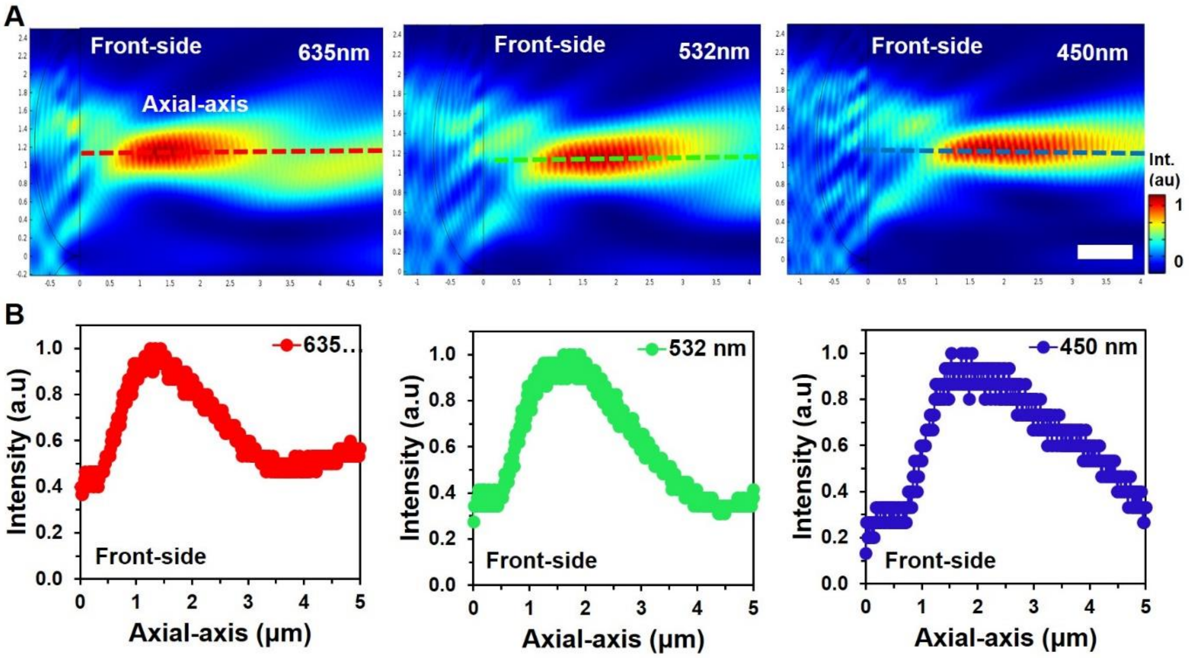


**Figure S4.** Numerical modeling front-side focusing (axial-axis) of FDMLs. (**A**) Simulation diagram of back-side focusing property of FDMLs and focused light intensity distribution through monochromatic (635, 532, and 450 nm) light at normal illumination. Scale bar = 2.5 μm. (**B**) Focused light bandwidth (FWHM) and intensity increased with incident wavelength.

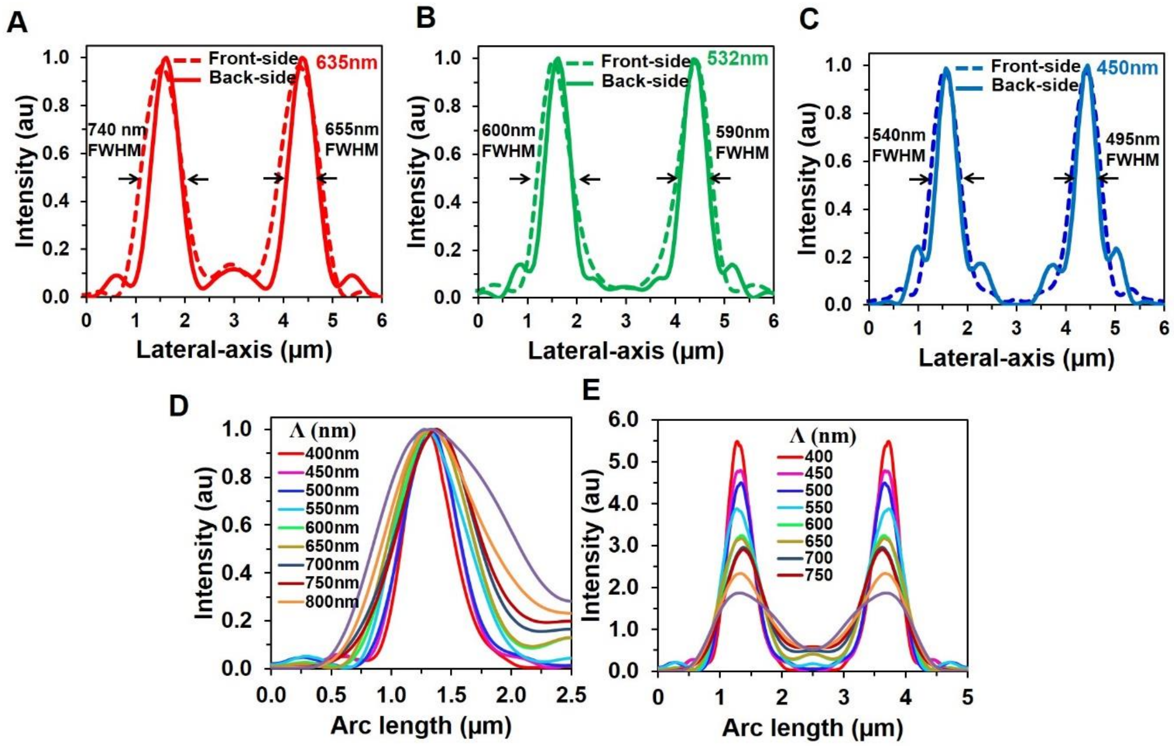


**Figure S5.** Front-side and back-side light focusing of FDMLs. **(A-C)** Simulation diagram of back-side focusing property of FDMLs and focused light intensity distribution through monochromatic (635, 532, and 450 nm) light at normal illumination. Scale bar = 2.5 μm. (**D, E)** Focused light bandwidth (FWHM) and intensity increased with incident wavelength.

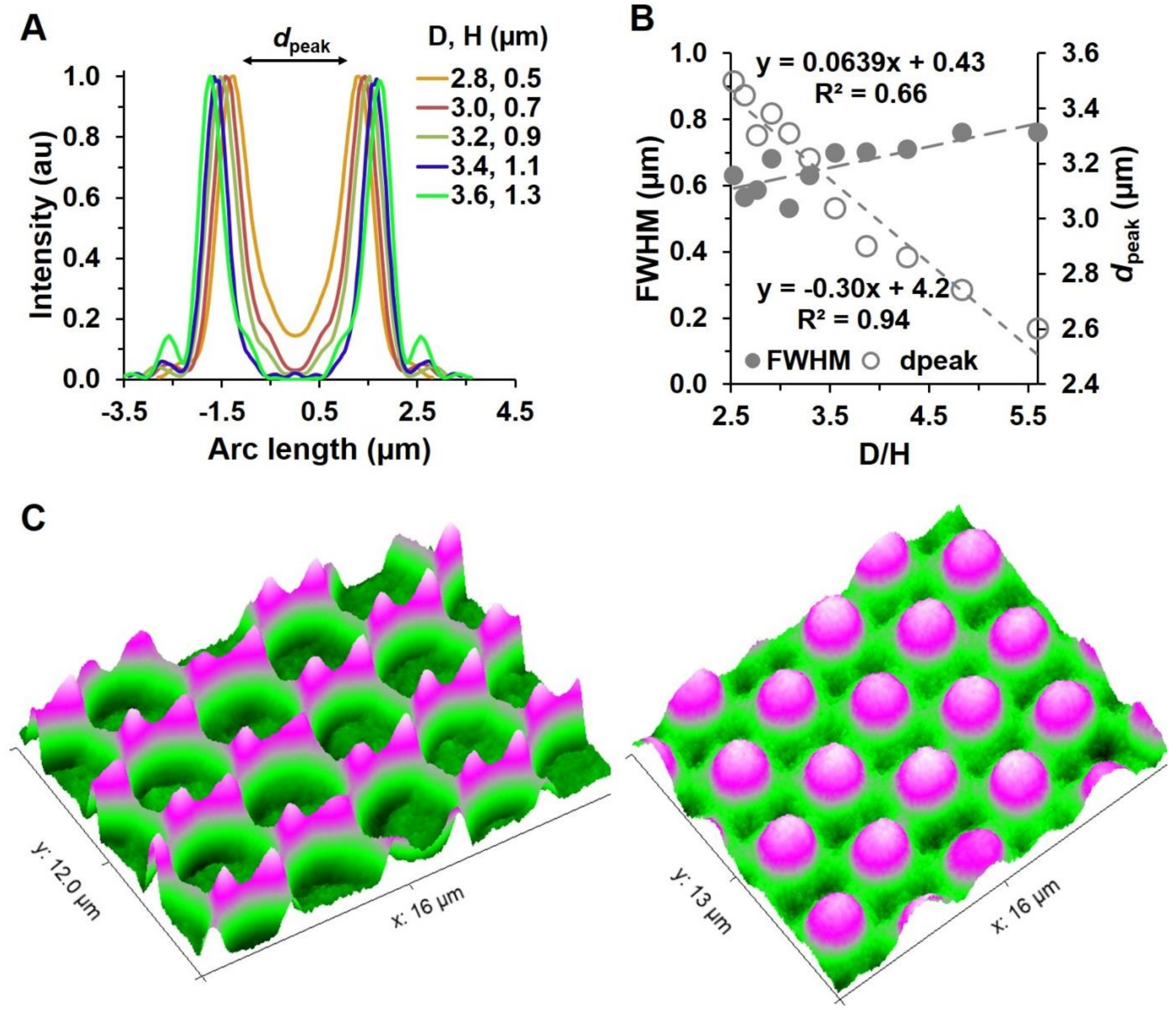


**Figure S6.** Back-side light focusing of FDMLs and 3D polarization sensitive hotspots. **(A, B)** The bandwidth **(**FWHM) and peak-to-peak distance ($d_{peak}$) of focused light as a function of width/height variation. (**C**) X- and Y-polarization sensitive 3D hot-spots areas during unfocused conditions.

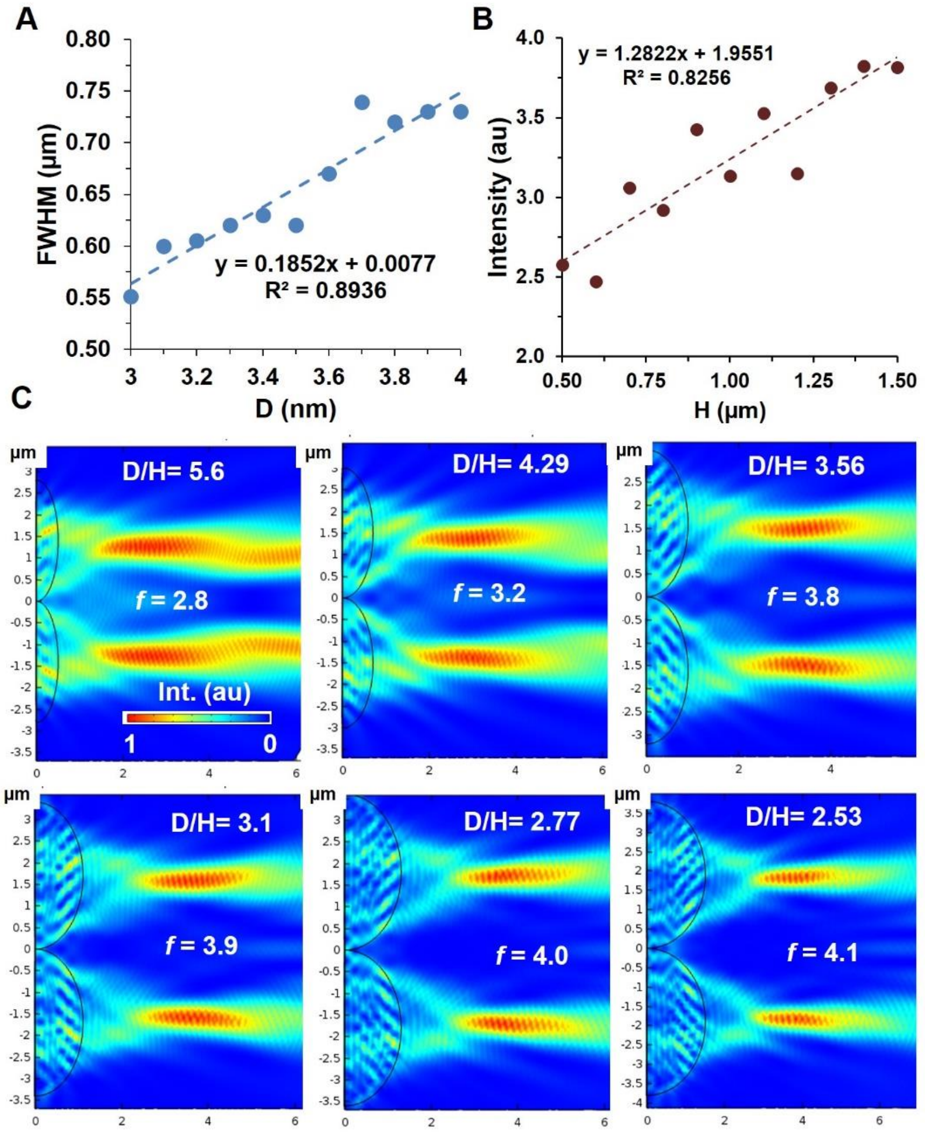


**Figure S7.** Back-side light focusing property of FDMLs and electric field distribution. (**A, B**) Focused light bandwidth **(**FWHM) and intensity through FDMLs diameter (**C**) and height (H) variations. (**C)** Focused light field distribution through D/H variations.

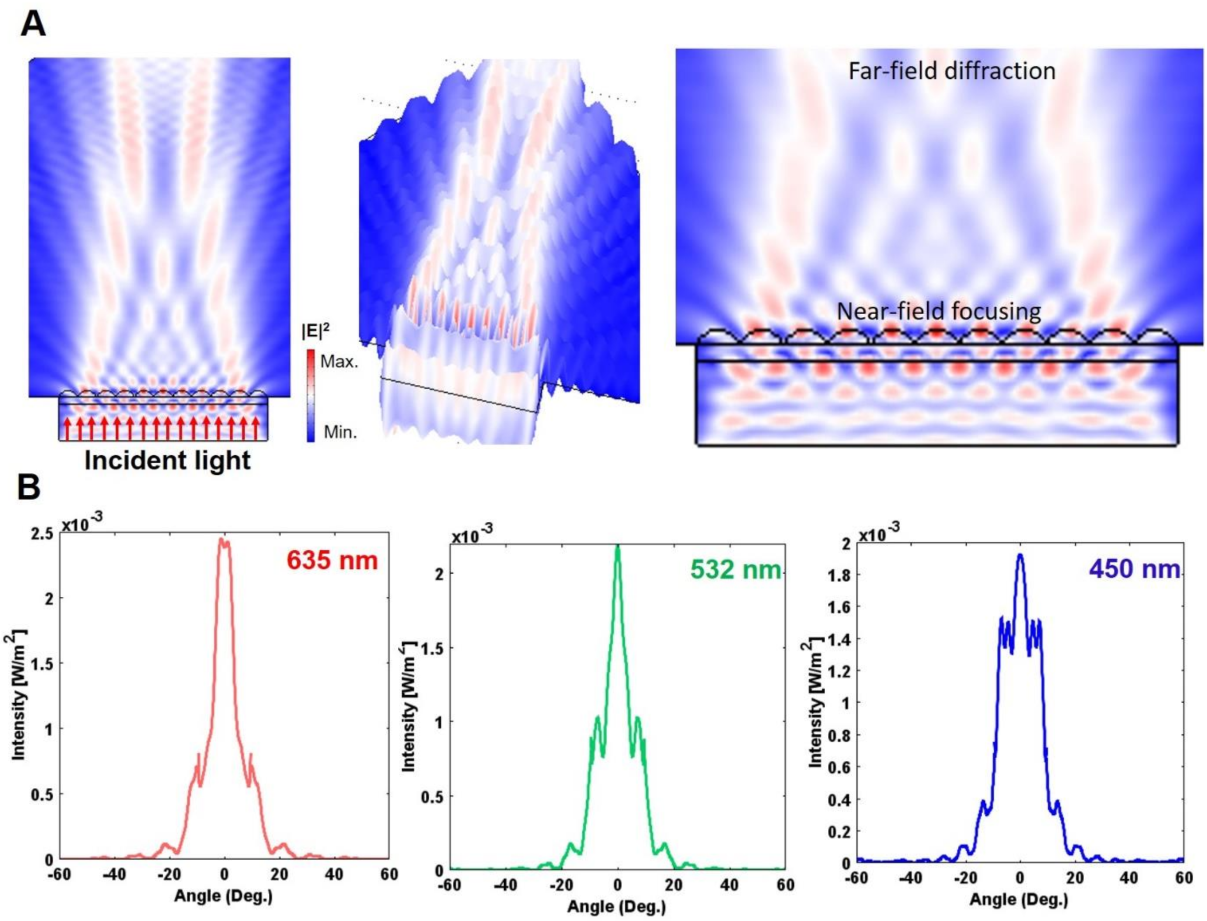


**Figure S8.** Near-field focusing and far-field diffraction modeling. (**A**) Computation modelling of near-field light focusing and far-field diffraction intensity distribution of FDMLs. (**B**) Far-field diffraction intensity distribution of illuminated monochromatic light (635, 532, and 450 nm) as a function of angle (degree).

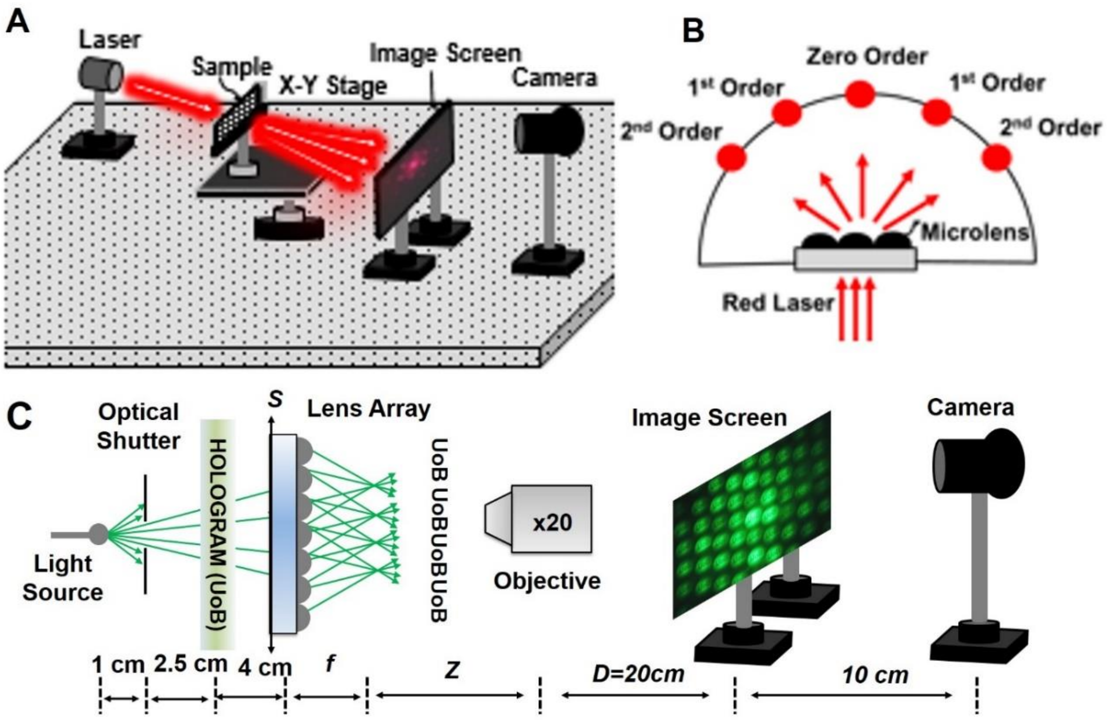


**Figure S9.** Far-field, near-field diffraction measurement and image project experiment setup. (**A**, **B**) Experiment setup for the far-field diffraction of FDMLs through an image-screen and transparent hemispherical surface. (**C**) A conceptual block-diagram of far-field projection experiment setup with applied stretch, *S*.

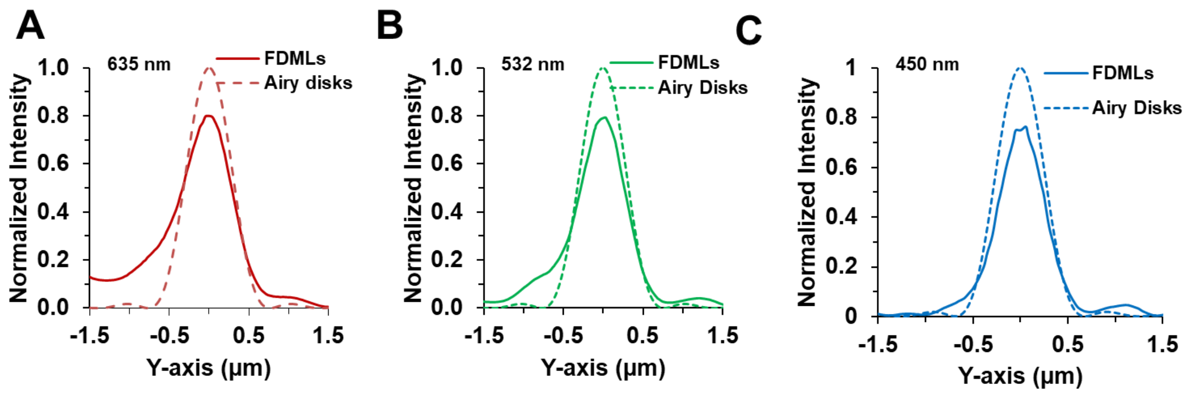


**Figure S10. Strehl ratios calculation for FDMLs.** (**A-C**) Measured focused light intensity of FDMLs and compared with ideal Airy discs profile. Strehl ratio is defined as the ratio between focused light intensity and theoretical diffraction-limited (theory) airy discs profile.[1] Therefore, calculated Strehl ratios of FDMLs are 0.8, 0.78, 0.77 for 635 nm, 532 nm and 450 nm, respectively.

## References

[1] M. Khorasaninejad, W. T. Chen, R. C. Devlin, J. Oh, A. Y. Zhu, F. Capasso, Science **2016**, 352, 1190.
